# Review of rearing-related factors affecting the welfare of laying hens

**DOI:** 10.3382/ps/pev123

**Published:** 2015-05-25

**Authors:** Andrew M. Janczak, Anja B. Riber

**Affiliations:** * Animal Welfare Research Group, Department of Production Animal Clinical Sciences, Faculty of Veterinary Medicine and Biosciences, Norwegian University of Life Sciences (NMBU), Oslo, Norway; † Department of Animal Science, Aarhus University, Tjele, Denmark

**Keywords:** laying hen, rearing conditions, animal welfare, behavior

## Abstract

Laying hens may face a number of welfare problems including: acute and chronic pain caused by beak trimming; exaggerated fearfulness that may cause stress and suffocation; difficulties in locating resources, resulting potentially in emaciation and dehydration; frustration and boredom, caused by an environment that is barren; feather pecking; cannibalism; foot lesions; and bone fractures. In Europe, a greater proportion of laying hens are housed in non-cage systems compared to the rest of the world. The extent of the different welfare problems may therefore vary between countries as the type of housing system influences the risk of suffering. More generally, many of these welfare problems are influenced by the rearing environment of the pullets. This article therefore focuses on welfare problems in laying hens that can be traced back to rearing. Factors that have been studied in relation to their effects on bird welfare include beak trimming, housing type, furnishing, enrichment, feeding, stocking density, flock size, sound and light levels, concentration of gasses, age at transfer from rearing to production facilities, similarity between rearing and production facilities, competence of staff, and interactions between bird strain and environment. The present review aims to summarize rearing-related risk factors of poor welfare in adult laying hens housed according to European Union legislation. It aims to identify gaps in current knowledge, and suggests strategies for improving bird welfare by improving rearing conditions. Two main conclusions of this work are that attempts should be made to use appropriate genetic material and that beak trimming should be limited where possible. In addition to this, the rearing system should provide constant access to appropriate substrates, perches, and mashed feed, and should be as similar as possible to the housing system used for the adult birds. Finally, young birds (pullets) should be moved to the production facilities before 16 weeks of age. The measures outlined in this review may be useful for improving the welfare of pullets and adult laying hens.

## INTRODUCTION

Consumers increasingly demand poultry products that are produced using high welfare standards. The 2012 ban on the use of conventional cages to house laying hens in the European Union, and the 2015 ban in California on selling eggs produced by hens in conventional cages, illustrate this trend. Ethical arguments based on a synthesis of utilitarianism, animal rights, and agent-centered views are increasingly accepted and support the view that animals should be protected from unnecessary suffering (Sandøe and Crisp, 1997). It is also becoming increasingly clear that research exploring and quantifying animals’ health and needs (Dawkins, 2008), combined with the ethical justification for various practices (Duncan and Fraser, 1997), can provide an educated basis for decisions regarding how we treat the animals in our care.

A large proportion of welfare problems in laying hens are influenced by the method by which pullets are reared from hatch until they are 15 to 18 weeks of age, after which they are moved from the rearing system to the laying system. Some problems may increase over time, mainly affecting welfare during the laying period, although a few problems are unique to the rearing period. It is well documented that early experience has long-term effects on the development of behavior, including unwanted abnormal behavior. Layer chicks start pecking and learning about appropriate food and pecking substrates during the first 24 h of life, as well as imprinting on conspecifics and developing fear-related avoidance of people and unfamiliar objects (Hess, 1959, 1964; Phillips and Siegel, 1966; Dawkins, 1968). Perch use starts during the first few days of life (Workman and Andrew, 1989; Riber et al., 2007a). This underpins the importance of the early rearing environment for the birds’ adaptation to the production environment which can affect appropriate foraging and pecking behavior, sensitivity to potential stressors, and ability the to navigate in a given production environment.

Development of welfare problems during rearing may be predictive of welfare problems during lay. For example, the plumage condition during lay is better when feather pecking had not started during rearing (Gilani et al., 2013; de Haas et al., 2014a). This is further supported by the observation that increased feather damage at 17 to 20 weeks of age is later associated with earlier onset of severe feather damage during lay (Drake et al., 2010). Seventy-one percent of rearing flocks with no feather damage continued without feather pecking problems during the laying period; whereas the risk of continued problems with feather pecking during lay is 90% for flocks in which feather pecking was recorded during rearing (Bestman et al., 2009).

Certain welfare problems such as beak trimming of one-day-old chicks, which causes acute pain (Gentle, 1986a), are unique to pullets, but most welfare challenges are interlinked and may impact birds both during the rearing and production phases. The rearing system may directly affect the welfare of the adult birds if the transition from the rearing system to the layer system induces fear, stress, emaciation, and dehydration. This is more likely to occur if the production system is very different from the rearing system. The consequences of some of the welfare problems, such as feather pecking, cannibalism, foot lesions, and bone fractures, may increase with age and be most apparent for adult layers. This is certainly the case for difficulties in navigating in a complex aviary system, which may result in the production of a high percentage of floor eggs, the manual collection of which incurs large costs. A systematic review of mortality in laying hens in aviaries indicated that genotype and access to litter during rearing accounted for the majority of mortality during production (Aerni et al., 2005). Similar findings have been reported for organic systems, where 79% of the variation in plumage condition between 23 organic farms was found to be due to rearing-related factors (Knierim et al., 2008). Appropriate rearing, as summarized here, is thus essential for ensuring the welfare and productivity of laying hens.

This review summarizes existing knowledge about rearing-related risk factors that influence the welfare of pullets and laying hens. Experimental, on-farm, and epidemiological studies are included. Factors during rearing that have been studied in relation to their effects on both the rearing and the adult phase include: effects of beak trimming, housing type, furnishing, enrichment, feeding, stocking density, flock size, sound and light levels, concentration of gasses, transition from rearing to production facilities, competence of staff, and the interactions between breed and environment. We aim to make recommendations for better rearing of pullets, but also to identify those topics that are not well understood, including a number of factors involved in rearing pullets for non-cage production systems.

## BEAK TRIMMING

### Legislation and Challenges connected with Beak Trimming

According to European legislation, beak trimming is permitted prior to 10 days of age (CEC, 1999: 1999/74/EC). In certain European countries, such as Norway and Sweden, beak trimming is prohibited by national legislation, and in Denmark the egg industry has decided to cease beak trimming. Beak trimming is performed primarily to reduce the incidence of feather pecking and associated cannibalism. Comparison of beak-trimmed and non-beak-trimmed birds in conventional cages and furnished cages suggests that beak trimming reduces mortality from 40 to 51% to 4 to 8% (Guesdon et al., 2006). It has also been suggested that beak trimming improves feather condition (Lee and Craig, 1991; Staack et al., 2007; Lambton et al., 2013), although one study found minimal feather pecking in both beak-trimmed and non-beak-trimmed birds (Sandilands and Savory, 2002a). Furthermore, a recent on-farm study found intact beaks to be a risk factor for severe feather-pecking during the rearing period, but not during the laying period (Gilani et al., 2013).

As a result of continual genetic selection, the genotypes used in today's egg production are likely to differ significantly from those used previously. Modern layer strains are potentially less susceptible to feather pecking and cannibalism, emphasizing the need for new studies investigating the efficacy of, and necessity for, beak trimming. Indeed, there are examples such as Norway, a country in which beak trimming is prohibited, which has a laying hen mortality as low as 2.26% in furnished cages and 3.69% in aviary systems (Hestetun, 2014). Stakeholders in other countries claim that feather damage in intact hens is still at unacceptable levels, but recent data to support this claim are lacking. Flock sizes in Norway are normally limited to 7,500 birds, whereas in countries such as The Netherlands a single producer may have three flocks of 30,000 birds each. Future studies are therefore called for in order to tease apart the effects of flock size, hybrid, management, housing, and rearing, on feather pecking in laying hens that are non-beak-trimmed. Without these studies, claims regarding the necessity of beak trimming seem poorly substantiated.

The beak contains thermoreceptors, nociceptors, and mechanoreceptors (Gentle, 1989). Partial amputation of the beak therefore results in pain, sensory loss, and a reduction in the bird's ability to manipulate objects (Gentle, 1986a). The beak is an important tool used by the bird in many activities including grasping food items, preening, removing ectoparasites, exploring the environment, nest building, and during agonistic interactions with other birds. Indeed, beak-trimmed birds have been found to have a higher infestation of ectoparasites due to a reduced ability to remove them (Mullens et al., 2010; Chen et al., 2011). The benefits of beak trimming should therefore be weighed against the costs.

### Acute Effects of Hot Blade and Infrared Beak-Trimming

When discussing the welfare implications of beak trimming, consideration should be made of the specific technique used, the age of the birds, and the severity of the treatment relative to acute and chronic effects. Two different beak-trimming methods are routinely used in Europe. Hot blade (**HB**) is the traditional method and can be performed at any age whereas infrared (**IR**) trimming is a more recently developed and more precise method that can only be performed at the hatchery. Unless stated otherwise, only results for birds trimmed at 10-day-old or younger are reported here.

The effects of HB trimming have been studied comprehensively. There appears to be a short pain-free period immediately after HB trimming which may last up to 26 h (Gentle et al., 1991; Glatz et al., 1992). After this period, trimmed birds not given analgesics show a significant reduction in feed intake compared with those given analgesics (Glatz et al., 1992). Both HB-trimmed and more severely IR-treated birds spend less time walking at 5 weeks of age than less severely IR-treated birds (Dennis and Cheng, 2012).

Both IR and HB trimming reduce feed intake relative to controls until 4 weeks of age (Marchant-Forde et al., 2008). Furthermore, both HB- and IR-trimmed birds were less active than controls up to one-week-old, with IR-trimmed birds having the largest proportion of the beak removed, being the least active (less time eating and drinking) and the controls being the most active (Marchant-Forde et al., 2008). Birds that are HB trimmed at 7 to 10 days change their meal patterns (Persyn et al., 2004). Beak-trimmed hens eat smaller meals and have shorter intervals between feeding than hens with intact beaks at 77 to 80 weeks of age. Another study indicates that HB beak trimming at one or 10 days of age reduces the activity of birds during the week following treatment, but not thereafter (Gentle et al., 1997). HB trimming at 2 days of age reduced the time spent pecking and the force of pecking relative to untrimmed birds at 3 weeks of age, but not at 4 or 5 weeks (Dennis and Cheng, 2010). Chicks that are beak-trimmed on the day of hatching do not appear to show a reduction in feeding or total pecking behavior at 10 weeks of age compared with controls, but the peak force used during exploratory pecks is significant less at 12 weeks of age (Jongman et al., 2008).

IR beak trimming causes a reduction in body weight from first weighing at 5 days of age that lasted until 8 weeks of age in trimmed compared with untrimmed chicks (Angevaare et al., 2012). These studies indicate that beak trimming is likely to cause acute pain in the immediate period following the procedure.

### Chronic Effects of HB and IR Methods Applied to Adults or Chicks

In the long term, beak trimming may also cause both chronic pain and reduced functionality of the beak. Intact birds eat more efficiently between 89 and 106 days of age, and pick up approximately 63% more food in a single peck than HB-trimmed birds (Duncan et al., 1989). Litter-directed behavior after HB beak trimming at 8 days of age is also performed less during the first 19 weeks of age in trimmed birds compared with intact controls (Sandilands and Savory, 2002b). Adult HB beak-trimmed hens display longer nesting durations, which may indicate pain and/or a reduced ability to manipulate nesting material (Eskeland, 1981). Although it is currently forbidden in the European Union, Norway, and Switzerland, beak-trimming after 4 weeks of age is still practiced in several other parts of the world and certainly causes long-term adverse effects on behavior, feeding activity, and weight gain (Duncan et al., 1989). Neuromas in the beak stump of birds that were beak trimmed as adults have been found 20 to 30 days after HB trimming one-third of the beak; and spontaneous firing of neurons has also been detected (Breward and Gentle, 1985). There is no evidence that HB beak trimming at 1 or 10 days of age causes neuroma formation (Gentle et al., 1997).

To summarize, chronic effects on bird welfare have mainly been found in birds that have been beak trimmed at one-week-old or later. Chronic pain due to IR trimming has to our knowledge not been reported, but this may be related to the fact that IR trimming is only performed at the hatchery on chicks less than 2-day-old. These studies indicate that beak trimming of birds older than one week is likely to reduce their welfare by causing chronic pain and inhibiting the normal expression of behavior.

### Severity of Beak Trimming

The severity of beak trimming, reflected in the amount of beak removed and the duration of the cauterization procedure, influences the likelihood of neuroma development as well as the development of chronic pain. If birds are HB trimmed with varying severity at hatch using different durations of cauterization, neuromas are found in the beaks of all trimmed birds at 10 weeks of age. The neuromas found in the mildest treatments regress, whereas those found in the severely trimmed beaks persist to 70 weeks of age (Lunam et al., 1996). More deformities are also observed on the beaks of the severely trimmed birds (Lunam et al., 1996). In addition to this, inappropriate temperatures may cause damage to the beak past the point of the cut (Gentle, 1986b). If practiced, beak trimming should therefore involve removal of the smallest possible portion of the upper beak.

### Comparison of HB and IR Beak-Trimming

A number of studies have directly compared the effects of HB and IR trimming on the behavior and welfare of chicks. HB-trimmed birds, and the more severely IR-treated birds, spent less time walking at 5 weeks of age than less severely IR-treated birds (Dennis and Cheng, 2012). HB-trimmed birds also spent less time drinking than IR-treated birds up to 10 weeks of age, which may be indicative of pain (Dennis and Cheng, 2012). IR-trimmed birds had better plumage than HB-trimmed birds (Dennis et al., 2009). Compared with HB-treated beaks, IR-treated beaks were more consistent in length (Carruthers et al., 2012) and had fewer abnormalities (cracks, asymmetrical regrowth, blisters, etc.) (Carruthers et al., 2012; Marchant-Forde et al., 2008). This study also indicated that both treatment techniques resulted in a reduction in feed intake relative to controls, with the IR-trimmed birds consuming the least food until 4 weeks of age. Furthermore, both HB- and IR-trimmed birds were less active than controls up to one week of age, with IR-trimmed birds being the least active (less time eating and drinking) and the controls being the most active. HB-trimmed birds, which had less of the beak removed, were intermediate in their activity levels. This comparison between the two trimming methods may however be confounded by the severity of the beak trimming, as the IR-treated birds were more severely trimmed than the HB-treated birds.

Another large study comparing IR- and HB-trimmed birds with intact birds indicated that the IR treatment reduced pullet body weight and feed consumption relative to the other treatments (Honaker and Ruszler, 2004). In addition, IR-trimmed pullets suffered higher mortality than HB-trimmed pullets. Thus existing results are to some degree contradictory, although the majority of studies find IR trimming to be a gentler method of beak trimming. Because beak trimming is likely to cause acute and chronic pain, and to detrimentally influence behavior, it should ideally be replaced by breeding, housing, and husbandry methods that make beak trimming unnecessary. The remainder of this review will focus on these latter possibilities for influencing laying hen welfare.

## REARING CONDITIONS AFFECTING THE WELFARE OF LAYING HENS

### Transfer from Rearing to Laying Environment

A study of 28 rearing flocks producing 51 flocks of laying hens indicates that earlier transfer of birds from the rearer to the producer decreases the severity of feather damage in adult birds, and leads to greater use of outdoor areas in organic production (Bestman and Wagenaar, 2003). Earlier transfer (16 weeks or less) of floor-reared birds to conventional laying cages that are modified by the incorporation of 2 nests also reduces the risk of laying outside of the nest area (Sherwin and Nicol, 1993). Similarly, birds remaining on the same farm during rearing and laying have a delayed onset of severe feather damage (Drake et al., 2010). In this case, an increasing number of differences between the rearing and the laying environments within the farm (feeder type, drinker type, light intensity etc.) were not shown to be associated with earlier onset of serious feather damage (Drake et al., 2010), possibly suggesting that environmental changes have a cumulative effect. Further research needs to be performed on the role of the transition from the rearing facility to the laying system as a risk factor for decreased welfare in the laying period.

### Transfer from Cages to Loose Housing Systems

There is general consensus that hens should be reared in an environment similar to that in which they will live as adults. Matching the rearing environment to the adult environment is thought to ease the transition to the layer house and reduce problems such as feather pecking and cannibalism, especially in non-cage systems (van de Weerd and Elson, 2006). The number of scientific studies in this area is however rather limited.

A range of welfare problems have been related to the combination of rearing in cages, followed by housing in aviaries during the laying period. The major concern about transferring pullets from cages to aviaries is their lack of experience with navigation in a 3-dimensional space. At 16 weeks of age, birds reared with access to perches from the time of hatching are more skilled at reaching the higher tiers by jumping from one tier to the next in comparison to birds who first gained access to perches at 8 weeks of age (Gunnarsson et al., 2000). Lack of ability to navigate in 3-dimensional space increases the risk of emaciation, dehydration, and floor eggs when food, water, perches, and nest boxes are located on the different levels found in aviary systems (Tauson, 2005). Flight accidents may be prevalent in birds reared without perches, resulting especially in keel bone fractures. Birds reared without access to perches lay more floor eggs and exhibit a higher incidence of cloacal cannibalism (Gunnarsson et al., 1999). Birds perching on the floor may be more susceptible to cannibalism because of a reduced ability of subordinate individuals to avoid aggressive pen mates (Cordiner and Savory, 2001; Yngvesson et al., 2002). Because of the significant problems associated with cage rearing followed by production in loose housing systems, to the authors’ knowledge, this is not commonly practiced. Practical experience suggests that certain hybrids may be more reluctant to go up into the aviary system than other hybrids, causing more floor eggs and associated problems. This illustrates the fact that some hybrids may need more training, irrespective of the system in which they are reared.

### Transfer from Loose Housing Systems to Cages

Loose housing systems provide birds with more opportunities to express natural behavior and may thus better fulfill their behavioral needs. However, if the pullets are destined for cages in the laying period, the transition may be associated with welfare problems. One of the concerns is that birds’ perceptions of space may be affected by their early experience (Faure, 1991). The general activity of floor-reared pullets following transfer to cages is reduced in the first days after transfer relative to cage-reared birds, likely reflecting the greater change that the floor-reared birds were thus exposed to (Craig et al., 1988). Another commercial scale study showed that aviary-reared birds exhibited more alert behavior near to a novel object than did cage-reared birds at 3, but not at 5, weeks after transfer (Tahamtani et al., 2014). Alert behavior in response to a novel object in the home environment is associated with a positive choice and therefore is an indicator of good welfare in laying hens (Nicol et al., 2009; Nicol et al., 2011). This finding suggests that the aviary-reared birds initially cope better with transfer from the rearing to production facilities than cage-reared birds (Tahamtani et al., 2014). However, mortality recorded throughout the production period was found to be higher in the aviary-reared than in the cage-reared birds, indicating long-term negative effects of aviary rearing for birds later producing in furnished cages.

At the end of lay at 68 weeks of age, hens reared in cages show more passive and less flighty responses to a human outside their home cage than floor-reared birds (Anderson and Adams, 1994a, using methods described by Jin and Craig, 1988; Hansen, 1976). Another study using the same recording method reported no effect on response to a person standing outside of the cage in 60- and 75-week-old hens reared in cages or floor pens and then housed in conventional cages during the laying period(Jin and Craig, 1988). The plumage condition was poorer at 60 but not at 75 weeks in the floor-reared birds. A similar study of cage- or floor-reared birds housed during the production period in furnished cages found floor-reared birds to have a poorer plumage at the end of lay (Roll et al., 2009).

These studies, with the exception of that of Jin and Craig (1988), yield similar results in suggesting that rearing in an unrestricted environment with access to a rich substrate, followed by transfer to a restricted environment with a relatively poor substrate, may cause frustration, resulting in damaging feather-pecking and deterioration of plumage. This problem may be exacerbated by the common practice of not providing enough scratching material in furnished cages. In addition, the pans or mats commonly used as scratching materials are not designed to limit loss of substrate but to maximize ease of cleaning. Substrate is therefore likely to be lost very quickly if it is used by hens.

Floor-reared birds transferred to furnished cages during the laying phase are more likely to lay eggs outside of the nest area and to be less stable in their choice of nest sites than cage-reared birds after transfer to modified conventional laying cages containing two nests (Sherwin and Nicol, 1993). Earlier transfer to laying cages reduces this effect however, and the time of transfer to the laying environment (16 weeks or earlier) has more influence on the numbers of floor eggs than the rearing environment alone. The use of dust baths in furnished cages has been found to be higher in floor-reared, as opposed to cage-reared, hens during the entire laying period (Roll et al., 2008). Indeed, cage-reared laying hens may not use dust-bathing substrate at all, as indicated by the accumulation of undisturbed materials in the litter pan in some furnished cages (A.M. Janczak, unpublished data).

Litter-reared birds housed in cages during the laying period have paler adrenal glands than birds reared in cages (Struwe et al., 1992). This suggests a lower chronic activation of the hypothalamic–pituitary–adrenal axis in birds reared on the floor. In contrast, a similar study indicated no effect of the rearing system on adrenal responsiveness at 50 or 70 weeks of age (Moe et al., 2010). The same study did, however, indicate that heterophil-to-lymphocyte ratios were significantly higher at 70 weeks of age in hens reared on deep litter and later moved to furnished cages, compared to those who were later moved to conventional cages (Moe et al., 2010). Conversely, antibody production in response to an immune challenge was greater in the hens that had been reared on litter (Moe et al., 2010). The authors suggest that the effects on immune response may have been associated with pathogenic load found in the floor and furnished-cage environments, rather than stress due to the rearing system or housing system.

Existing studies thus suggest that hens should be reared in an environment similar to the one in which they will live in as adults.

### Transfer between Loose Housing Systems

There are several different designs of loose housing system. In the European Union, there is a trend away from floor systems in favor of multi-tier aviaries for both rearing and production phases. This trend is partly a result of the fact that these systems facilitate better utilization of available space in the shed, and partly a result of producers’ experience that aviaries work better than older floor systems. Aviary-reared birds have been found to use aviary platforms on which feed troughs, water nipples, perches, and nest boxes are normally located, and to fly and jump more often than floor-reared hens throughout the laying period (Colson et al., 2005). They also lay fewer eggs outside of nests, potentially reducing the risk of cloacal cannibalism (Colson et al., 2005). Rearing in aviaries with feed and water provided on elevated platforms (aviary rows), when compared with provision of feed on the floor, increases the use of elevated aviary levels at adulthood, results in a higher accuracy of long flights and jumps, reduces mortality during rearing, and increases the number of eggs laid in nest boxes (Colson et al., 2008). Indeed, the mortality rate of floor-reared hens is higher than that of aviary-reared hens with feeders located on platforms both before and after transfer from the rearer to the producer (Colson et al., 2008). Aviary-reared birds are also better at solving spatial tasks, as indicated by their better working memory in hole-board tasks (Tahamtani et al., 2015). They have bones with better load bearing capability and stiffness (Regmi et al., 2015) compared to cage-reared birds. It has thus been clearly demonstrated that hens destined for aviaries should be reared in aviaries.

### Effects of Furnishing and Enrichment

Birds may be especially sensitive to environmental effects during the early sensitive periods (Bateson, 1979). Early experience with furnishings and enrichment materials may therefore have long-lasting consequences (Johnsen et al., 1998), and exposure to appropriate enrichment experiences during the rearing period may reduce the risk of abnormal behavioral development. For example, feather pecking is thought to develop because of a lack of experience with foraging or dust-bathing material, resulting in ground pecks being redirected to the feathers of conspecifics (Hoffmeyer, 1969; Blokhuis and Arkes, 1984; Vestergaard et al., 1993; Vestergaard and Lisborg, 1993). The sensitive period for learning about food and dust-bathing material is during the first 10 days of life (Brown, 1964; Hess, 1964; Sanotra et al., 1995; Vestergaard and Baranyiova, 1996). In addition, rearing in a complex environment increases the chances that the birds will develop the necessary skills to navigate in a complex environment (Gunnarsson et al., 2000); and early exposure to varied stimulation may reduce later fearfulness (Jones, 1982; Reed et al., 1993). Documented positive correlations between fearfulness and feather pecking (de Haas et al., 2014a; b) suggest that early exposure to environmental variability may also reduce the risk of developing feather pecking.

### Perches

Pullets are highly motivated to perch and prefer to use the highest perches available, suggesting that this anti-predator behavior is highly conserved despite many generations of domestication (Newberry et al., 2001). Depending on perch height, chicks begin perching between 7 and 10 days of age (Workman and Andrew, 1989; Riber et al., 2007a). The amount of time spent perching increases steadily over time and there are positive correlations between early daytime perch use and later night time perch use in connection with roosting (Heikkilä et al., 2006). It is well documented that adult perch use is influenced by access to perches during rearing (Faure and Jones, 1982; Appleby et al., 1983), suggesting that learning to use perches is slower after the rearing period and may become permanently impaired (Gunnarsson et al., 2000). Whereas the physical process of jumping up onto perches (that is, the motor mechanism) is thought to be prefunctionally developed in chicks, they have to learn to move in more than 2 dimensions (Appleby and Duncan, 1989). Thus the perceptual mechanism for recognizing the perches as possible resting or escape routes requires functional experience to develop (Appleby and Duncan, 1989). A positive association between first perch use and time spent under perches during the first 2 weeks post-hatch supports this argument (Heikkilä et al., 2006). The importance of early experience with perches has been highlighted in many studies. A more complex rearing environment produces birds that demonstrate greater use of elevated aviary levels, higher accuracy of long flights and jumps, lower pullet mortality, and a higher proportion of eggs laid in nest boxes during adulthood (Colson et al., 2008).

A study of 59 Swedish flocks of different hybrid showed that access to perches during rearing reduces the prevalence of both floor eggs and cloacal cannibalism in loose-housed birds during the laying period (Gunnarsson et al., 1999). Another study of 64 Swiss flocks indicated that rearing with access to perches also reduces the occurrence of feather pecking during the rearing period (Huber-Eicher and Audigé, 1999). Impaired development of perching, associated with increased fearfulness and resulting in poor usage of perches, may increase the risk of smothering caused by panic and the piling up of birds into heaps (Hansen, 1976; Brake et al., 1994; Keeling, 1997). Furthermore, access to perches during rearing results in fewer broken back claws, improved bone mineral content, and improved bone strength in hens later housed in conventional cages as adults (Hester et al., 2013a,b). This is important because keel bone damage in adult birds is correlated with poor bone strength (Rodenburg et al., 2014). Existing studies thus suggest that pullets should be provided with perches throughout rearing if they are expected to use perches as adults.

### Substrate and Its Quality

Early experience with litter has significant effects on the development of pecking behavior, as indicated by a large number of experimental studies. The provision of sand and straw compared with wire from hatch until 4 weeks of age results in increased plumage quality, reduced feather pecking, reduced expression of fear-related behavior in a tonic immobility test, and reduced mortality in adult birds later housed on straw (Johnsen et al., 1998). This is consistent with an earlier study indicating that rearing on substrate instead of wire results in less feather pecking in adult birds housed in pens (Blokhuis and van der Haar, 1989) and has been confirmed by a later study showing that a lack of substrate in the first 4 weeks of life is related to increased feather pecking in adulthood (Bestman et al., 2009). Rearing chicks on litter or sand as opposed to wire has a preventive effect on the development of feather pecking even when the hens are later moved to a barren environment (Vestergaard et al., 1997). Beneficial effects on feather pecking may be particularly clear after the provision of long straw (Huber-Eicher and Wechsler, 1998; Blokhuis, 1991), but the provision of sand and peat during rearing also reduces plumage deterioration in adult birds (Nørgaard-Nielsen et al., 1993).

There is also ample documentation on the importance of early experience with litter from large on-farm studies. In aviary systems, early access to litter lowers mortality (Aerni et al., 2005) and access to wood-shavings for a minimum of 10 days during the rearing period reduces feather pecking compared with keeping hens continually on wire throughout the rearing and adult periods (Nicol et al., 2001). The latter study however indicated that the current substrate was of great importance, and adult hens housed on wood-shavings performed significantly more ground pecking and less feather pecking than birds housed on wire, regardless of previous experience. A Swiss study used data from aviary-reared birds housed on plastic grids or having access to litter in rows for the first 2 weeks of life (Huber-Eicher and Sebö, 2001). All groups were later housed under identical conditions with unrestricted access to litter. The results indicated that birds reared on litter spent more time foraging at 5 weeks of age and less time feather pecking at 5 and 14 weeks (Huber-Eicher and Sebö, 2001). The first observation is crucial, as a higher proportion of hens foraging is associated with a lower incidence of severe feather pecking during the rearing period in organic and indoor loose housing systems (Gilani et al., 2013). A recent study of 47 rearing flocks in the Netherlands showed that disruption of access to litter from 7 to 10 days post-hatch, and limitation of litter supply in the aviary rows, increases the incidence of severe feather pecking and feather damage, and increases fearfulness during the rearing period (de Haas et al., 2014b). This illustrates the previous argument that moving birds from a system with a better to a poorer substrate quality may be instrumental in causing the development of damaging feather pecking behavior. The study by de Haas et al. (2014b) is striking in indicating significant benefits of substrates as simple as paper soiled with feces and feed. In a study of 28 commercial organic flocks in the Netherlands, the absence of litter during the first 4 weeks of rearing was predictive of an elevated incidence of feather pecking during the laying period (Bestman and Wagenaar, 2003).

The importance of early developmental processes in determining the incidence of feather pecking is emphasized by positive associations between measures of severe feather pecking as early as 5 weeks of age, fear of humans during rearing, and feather damage at 40 weeks of age in aviary-housed laying hens in conventional systems (de Haas et al., 2014a). It is emphasized that even relatively brief interruptions in access to a substrate during rearing may have detrimental consequences for birds that normally have access to substrate (de Haas et al., 2014b).

### Outdoor Access during Rearing for Organic Systems

Only a few studies have tested the effects of access to outdoor areas during rearing. This may be important because usually only a small proportion of adult hens use outside ranges in organic systems at any particular time (Bubier and Bradshaw, 1998; Zeltner et al., 2004; Hegelund et al., 2005; Keeling et al., 1988). It is likely that early experience with outdoor areas during rearing influences the use of outdoor areas in adult birds. Birds that were both handled and exposed to an outdoor area from 12 to 20 weeks of age emerged more quickly from a test box placed outside, and also moved further away from the box, than birds that were only handled or were neither handled nor exposed to an outdoor area (Grigor et al., 1995). A more recent study indicates that 6-week-old layer chicks exposed to one week of access to an outdoor area were less fearful and learned to find a food reward significantly faster than controls without outdoor access (Krause et al., 2006). Taken together, these studies suggest that early exposure to an outside area during rearing should increase the use of outdoor space by adult hens in organic production.

### Enrichment, Brooders, and Noise

There is evidence that the provision of enrichment during rearing reduces fearfulness in both pullets and adult laying hens. For example, birds reared in pens containing brightly colored plastic bottles, balls, and rattles, and exposed to human voices from a radio during daylight hours, as well as human handling, had lower levels of potentially damaging fear reactions and incurred fewer knocks against the cage during depopulation than non-enriched birds (Reed et al., 1993). Chicks raised with a variety of objects are reported to be more mobile and to feed and vocalize more in open field tests. They also take less time to emerge into an open area in hole-in-the-wall tests (Jones, 1982) than birds without an enriched environment. Access to environmental enrichment in the form of peckable strings during the rearing period has been shown to reduce feather pecking in both the rearing and the laying periods, with the effect on the rearing period being most pronounced if the objects were introduced from the time of hatching (McAdie et al., 2005). In contrast, the introduction of strings during the laying period is ineffective for reducing feather pecking in adult laying hens (Glatz, 2000).

Dark brooders are warm, dark, and enclosed areas that may simulate the effects of a brooding hen. Four-week-old chicks reared in dark brooders expressed lower levels of severe feather-pecking than those brooded under heat lamps in experimental conditions (Johnsen and Kristensen, 2001). Dark brooders were also found to have long-term preventive effects on severe feather-pecking and cannibalism in an experimental study (Jensen et al., 2006). At 23 weeks of age, hens with intact beaks reared in brooders had better plumage and skin condition, and reduced mortality compared with control birds. Data collected from a large on-farm study found a reduced prevalence of severe feather-pecking and improved plumage condition in intact birds over the period from brooder placement to 35 weeks of age, with no adverse effect on growth, body-weight uniformity, or mortality to the end of rearing (Gilani et al., 2012). Dark brooders are also reported to improve behavioral synchrony between birds, reduce disturbances during resting, and result in calmer birds (Riber et al., 2007b; Gilani et al., 2012).

Whereas exposure to audio stimulation from a radio has beneficial effects, this does not apply to high sound levels in general. A study of 34 flocks from 29 rearing farms in the UK indicates that the probability of severe feather-pecking at 35 weeks is increased when the range of sound levels in the house at the end of rear is high (mean 7.8 dB, range 0 to 18 dB; Gilani et al., 2013). Furthermore, higher average sound levels during rearing (mean 58.3 dB, range 32 to 66 dB) are associated with increased feather damage at 35 weeks of age (Gilani et al., 2013). This is corroborated by another study of 22 free-range and organic laying farms which identified higher sound levels during rearing (mean 59.4 dB, range 14.3 to 80.0 dB) as risk a factor for the earlier onset of severe feather damage (Drake et al., 2010). Between 15 and 17 weeks of age, each 10 dB increase in sound level was associated with a 25.5% reduction in the time to reach a cutoff point at which the average plumage condition of the flock was defined as poor, and between 17 and 20 weeks, with a 7.9% reduction in the time to reach the cutoff point for poor plumage condition (Drake et al., 2010). Investment in the development of quieter systems is therefore likely to be beneficial.

### Effects of Feeding-Related Factors

An experimental study has suggested that pullets fed pellets have more plumage damage than pullets fed mashed feed (Savory et al., 1999). Other experimental studies suggest that provision of whole grain in the substrate during the rearing period increases foraging directed to the floor, and reduces damage due to feather pecking in adult birds (Blokhuis, 1991; Blokhuis and van der Haar, 1992). A study of 34 flocks from 29 rearing farms indicates that an increasing number of diet changes during rearing increases the incidence of feather-pecking outbreaks in adult birds (Gilani et al., 2013). A study including 22 free-range and organic laying farms (Drake et al., 2010) indicated that the presence of chain feeders, compared with feed hoppers, was associated with an earlier onset of severe feather damage. This could possibly be explained by higher feed intake in pullets fed using a chain feeder as increased feed intake in pullets is also associated with a risk of earlier development of severe feather damage (Drake et al., 2010).

Few studies have addressed effects of the feeder space provided during rearing. Existing studies were conducted more than 20 years ago (Anderson and Adams, 1994a,b) and may therefore no longer be valid, as the genetic lines used today are different and for example, body size and feed intake have changed (Anderson and Jones, 2012). However, one recent study reports that the rate of severe feather-pecking is lower when more than one feeder type is used, and higher when compartmentalized pans are used, as opposed to the sole use of chain feeders (Gilani et al., 2014). In summary, the provision of mashed or crumbled feed rather than pellets is one approach to reducing the risk of feather pecking.

### Effects of Stocking Density and Flock Size

European Union legislation allows a maximum density of 18 birds/m^2^ during rearing in barren cages, and 13 birds/m^2^ during rearing in furnished cages (CEC, 1999, Council Directive, 1999/74/EC). No maximum stocking density is defined for birds reared in aviary systems. There are few studies testing the effects of stocking density and flock size during rearing. A few studies were performed more than two decades ago (Anderson and Adams, 1992; Carey, 1987), but it is difficult to apply these results today as modern hens are different from the lines used at that time, and housing conditions have also changed. Results from earlier studies of stocking density must also be interpreted with care as in most studies, especially the older ones, stocking density was altered by adjusting the number of birds in a cage or pen, thereby also affecting feeder and drinker space allowances.

A study of 64 Swiss flocks indicated that rearing at a lower stocking density (<10 pullets/m^2^) reduces the occurrence of feather pecking in adults (Huber-Eicher and Audigé, 1999). Hansen and Braastad (1994) found that rearing at 6.5 pullets/m^2^ rather than 13 pullets/m^2^ reduces the occurrence of feather pecking during rearing, but not during the laying period. However, the plumage condition was better in the laying period in hens reared at the lower density.

Similarly, a Dutch study of 28 rearing flocks, split into 51 laying-hen flocks, indicated that a higher number of pullets per square meter (range 15 to 53 pullets/m^2^) in the first 4 weeks of life was associated with an increased incidence of feather damage during the rearing period (Bestman et al., 2009). However, the researchers noted that it was difficult to differentiate the influence of stocking density from the influence of a lack of litter during the first 4 weeks, as most of the high-density flocks also lacked litter during this period. In contrast, another comparable on-farm study indicated no association between stocking density during rearing and feather pecking during the rearing or production phases (Gilani et al., 2013). Spindler et al. (2013) used a planimetric method to compare the effects of space allowance in alternative housing systems for laying hens. The floor space covered by the pullets was determined using a software program calculating the animal area from color contrast photographs of standing and sitting pullets. This was done at regular intervals from the 6th week of life until the pullets were 18 or 20 weeks old. A correlation between floor space covered by the pullets in the standing position and the live weight was found. The authors concluded that the maximum stocking density for pullets at the age of 16 weeks should be between 11 and 14 birds/m^2^, depending on the genetic line used (Lohmann Traditional: 11 birds/m^2^; Lohmann Brown: 12 birds/m^2^; Lohmann Selected Leghorns: 13 birds/m^2^; and Dekalb White: 14 birds/m^2^). However, they also recommended that these suggested maximum stocking densities should be verified by further investigations using observations of behavior.

The few existing studies testing the effects of flock size involve flocks that are significantly smaller than those used on commercial farms today (Meuniersalaun et al., 1984). Stocking density, total area, and enrichment are thought to have a greater effect on welfare than flock size. An exception is found for the effects of flock size on the use of outdoor areas. The percentage of pullets that use an outdoor range is higher for smaller flocks (flock size range 92 to 15,848; Gilani et al., 2014), and this corresponds to findings for laying hens (Bubier and Bradshaw, 1998; Zeltner et al., 2004; Hegelund et al., 2005).

### Effects of Lighting

Older studies suggest that high light intensities during rearing results in pullets with a poorer plumage condition (e.g. see Hughes and Duncan, 1972). Similar results from more recent studies also indicate that high light intensity during rearing (30 lux vs. 3 lux) increases the prevalence of severe feather-pecking at 10 and 45 weeks of age, but not at 28 weeks of age (Kjaer and Vestergaard, 1999). In this study, the increased incidence of severe feather-pecking negatively affected plumage condition at 11 weeks of age, but not at 28 or 46 weeks of age. High light intensity during rearing also tends to have a long-term negative impact on mortality from 16 to 46 weeks (Kjaer and Vestergaard, 1999). Smaller differences in light intensities (3 lux or 10 lux) do not appear to influence the development of feather pecking or cannibalism (Kjaer and Sørensen, 2002). Another study indicates that a high light intensity (5 lux vs. 60 to 80 lux) during rearing does not influence the incidence of cannibalism during the pre-laying period or the early laying period (Hartini et al., 2002).

A study of 22 free-range and organic laying farms identified high light intensities during rearing as a risk factor for the earlier onset of severe feather damage; each 100-lux increase was associated with a 12.2% reduction in latency to reach a cutoff point where the average plumage condition of the flock was defined as poor (Drake et al., 2010). In contrast, the absence of daylight at the age of 7 to 17 weeks, in combination with not having litter at the age of 1 to 4 weeks, was found to be a significant predictor of feather damage during the laying period in an on-farm study run in The Netherlands (Bestman et al., 2009). Another recent study compared the percentage of loose-housed flocks with or without plumage damage according to whether they were exposed to daylight through windows (23 flocks) or not (58 flocks; Yngvesson et al., 2011). Thirty percent of flocks with windows had damaged plumage compared to 50% of flocks not exposed to daylight, suggesting that daylight exposure is not associated with increased risk of plumage damage.

Gentle feather-pecking may increase in pullets reared with low light intensities (3 lux vs. 30 lux), suggesting that low light intensity may impair the ability to identify environmental cues (Kjaer and Vestergaard, 1999). This may also explain why Gilani et al. (2013) found that a larger variation in light intensity within the house was associated with a reduced frequency of gentle feather-pecking during rearing. A light intensity above 5 lux is necessary for proper inspection of birds by the farmers. At intensities below 5 lux, light does not pass directly through the skull and cranial tissues to the pineal gland, where it would normally suppress the production and release of serotonin and melatonin (Zawilska et al., 2004). This is important, because melatonin modulates growth in poultry (Zeman et al., 1999), and low serotonin levels may increase feather pecking (van Hierden et al., 2002, 2004), aggression (Shea et al., 1990), and fear (Newberry and Blair, 1993).

A comparison of farms providing photoperiods ranging from 9.8 to 24 h/d indicates that the risk of severe feather-pecking during rearing, but not laying, is increased with shorter photoperiods (Gilani et al., 2013). The length of the photoperiod may also indirectly affect the prevalence of cloacal cannibalism. An onset of lay prior to 20 weeks of age has been related to an increased risk of vent pecking (Potzsch et al., 2001). Since the timing of sexual maturation can be controlled by using short photoperiods followed by increasing day length (Lewis et al., 1998), the risk of cloacal cannibalism can potentially be reduced by using a lighting schedule that delays the onset of lay. Birds reared with 23 h of continuous light and 1 h of darkness are reported to react less fearfully in a tonic immobility test, and to be easier to handle than those reared with a lighting program that changes from 6 to 23 h of light depending on the age of birds (Newberry and Blair, 1993). Another study indicates that chicks reared in 16 h of light have a higher lymphocyte count and a more active lymphocyte response than chicks reared in 8 h of light, indicating an enhanced immune response in birds exposed to a longer photoperiod (Mashaly et al., 1988).

Other aspects of light may potentially affect the welfare of pullets. The ability of adult laying hens and broilers to detect flickering depends on the color temperature of the light and the light intensity although detection of flickering above 105 Hz has not been documented (Nuboer et al., 1992; Jarvis et al., 2002). Even when poultry have been shown to perceive flickering, few studies indicate that this is aversive to birds (see review by Prescott et al., 2003). The light source and spectrum, or color, of the light are also important features. Some studies have shown a preference for alternatives to incandescent lighting (biolux lighting, Kristensen et al., 2007; fluorescent lighting, Widowski et al., 1992); however incandescent lighting is in the process of being phased out in favor of more energy-efficient lighting. Light-emitting diodes (**LED**) are thought to be the light source that will be most commonly used in poultry houses in the near future.

Research on LED and other light sources has mainly been concentrated on broilers, and positive effects of LED have been found on performance and welfare (Mendes et al., 2013; Riber, 2014). One useful feature of LED is that they are produced in all monochromatic colors and all possible polychromatic color temperatures. It is therefore possible to adjust the spectrum of the light to meet the birds’ preferences. Studies on the effects of flickering, different light sources, and different color temperatures on pullet welfare are limited (but see Gunnarsson et al., 2008a). It is, however, likely that the findings for adult birds are applicable to pullets.

In pullets reared for a laying system with access to an outdoor range, the light conditions during rearing may affect the use of the outdoor area, both during rearing and laying. The percentage of the flock observed on the range during the combined rearing and laying period increases with a higher light intensity in the house (mean 123 lux, range 2 to 687 lux; Gilani et al., 2014). Pullets reared for a production system with access to outdoor areas should be reared with access to natural light, as pullets reared with access to natural light show a higher preference for natural light at the age of 14 weeks than pullets reared without access to natural light (Gunnarsson et al., 2008a). In addition, pullets reared with access to natural light tend to start nighttime perching at an earlier age, which may reduce the prevalence of cannibalism (Gunnarsson et al., 2008b).

### Gas Concentrations and Air Quality

A large on-farm study indicates that elevated concentrations of carbon dioxide (range 43 to 2000) and ammonia (range 0 to 100 parts per million (**ppm**)) are risk factors for the earlier onset of severe feather damage (Drake et al., 2010). In this study, between the ages of 15 and 17 weeks, every 15 ppm increase in NH_3_ recorded was associated with a 10.1% reduction in the time taken to reach a threshold score for severely damaged plumage. A lower ceiling height in the rearing house (range 0.7 to 3.1 m) is associated with a higher prevalence of severe feather-pecking during rearing, but not during lay (Gilani et al., 2013). This latter finding could be related to increased ammonia and carbon dioxide concentrations resulting from poorer ventilation in poultry houses with low ceilings.

### Effects of Staff Competence

Lambton et al. (2013) in the UK tested the protective effects of a farm management package aimed at reducing the development of damaging pecking in loose-housed laying hens. Fifty-three treatment flocks, in which the producers were advised in the use of preventative strategies against feather pecking, were compared with 47 that were not instructed in the use of preventative measures. The study revealed that the implementation of the management package, including measures directed at the rearing period, significantly reduced levels of injurious pecking during the laying phase (Lambton et al., 2013). Another study of 34 UK flocks from 29 rearing farms supported the interpretation that the degree of experience of the staff caring for birds during the rearing period contributed significantly to a reduction in feather pecking during lay (Gilani et al., 2013).

Managing growth to avoid the production of underweight pullets reduces the risk of uterine prolapse and hence cloacal cannibalism, as uterine prolapse may trigger cloacal cannibalism (Glatz, 2005). It is also commonly thought that avoiding underweight pullets, and increasing uniformity, may lower the risk of feather pecking and cannibalism. However, research into this area seems to be very sparse. One study of Danish rearing flocks (both conventional and organic) showed that the lower the mean weight at 15 weeks of age in flocks of both white (*n* = 222) and brown strains (*n* = 158), the higher the mortality in the rearing period (Mørch et al., 2012). For the brown strains, uniformity of the flock also affected mortality; the less uniform flocks having the highest mortality in the rearing period. A tendency was found for flocks (*n* = 39) of Lohmann Selected Leghorn (LSL) hens housed in cages (conventional cages and furnished cages) with a uniformity above 90% at 15 weeks of age to have a lower mortality during the laying period than flocks with uniformity between 85 to 90% or below 85%. In contrast, the body weight at 15 weeks of age had no effect on mortality during the laying period (Mørch et al., 2012).

### Effects of Bird Hybrid and Interactions with the Environment

The genetic makeup of the birds may interact with environmental parameters and thereby influence bird welfare during both the rearing and laying periods. Strains may differ in their propensity to perform injurious pecking or to express exaggerated fear responses. Genetic selection against feather pecking may be a viable and more ethically acceptable alternative to beak trimming.

Genetic selection against abnormal behavior has been shown to be possible with regards to both feather pecking (Su et al., 2005) and cannibalism (Craig and Muir, 1993; Muir and Craig, 1998). Both types of behavior are heritable and can be reduced using breeding. The heritability of feather pecking has been estimated to range from 0.09 to 1.04, depending on the selection method and variable measured (reviewed by Jensen et al., 2008); whereas a single study reports a heritability for cannibalism of 0.65 (Craig and Muir, 1993). Breeding companies apparently select against feather pecking and cannibalism but the specifics are not published. Experimental lines of laying hens differing in their propensity to feather peck have been created using individual selection, but these lines have been used only for experimental research (Kjaer et al., 2001).

Selection for reduced fearfulness, which can be defined as the propensity to express fear reactions to dangerous or potentially dangerous stimuli, is complicated by its multifactorial nature and the plethora of methods available for measurement. Fear responses may also be stimulus-specific, meaning that selection for responses to one stimulus may be unrelated to responses to other stimuli. Furthermore, a bird may show active fear responses in the form of panicky flight, or passive fear responses in the form of tonic immobility. Tonic immobility as measured in a tonic immobility test (one example of a standardized fear test) has a heritability ranging from 0.18 to 0.32 (Craig and Muir, 1989; Campo and Carnicer, 1993).

Some studies indicate breed differences in fearfulness. For example, two lines of commercial floor-reared pullets subjected to several fear tests at 5 different times from hatching to 30 weeks of age illustrated strain-by-age interactions in response to the tonic immobility test (Hocking et al., 2001). Albentosa et al. (2003) obtained mixed results when comparing responses of four different strains in three different fear tests. Strain did not influence behavior in the novel object test or the open field test but White Leghorns reacted more fearfully than the other strains in the tonic immobility test. Two studies indicate a lack of differences in fearfulness between different genetic stocks (Hocking et al., 2004; Anderson and Jones, 2012). The variation within lines may be large and therefore selective breeding against fear within lines could be beneficial. Group housed birds selected against mortality associated with feather pecking were more active in an open field test at 5 to 6 weeks of age than randomly selected control birds (Rodenburg et al., 2009). Higher activity in an open field test indicates lower levels of fearfulness and this result therefore suggests positive associations between fearfulness and feather pecking that could be utilized in selection programs (Rodenburg et al., 2009). De Haas et al. (2014a) also found positive associations between measures of severe feather pecking as early as 5 weeks of age and fear of humans during rearing in aviaries, emphasizing that the relationship between fearfulness and feather pecking is not experiment-specific. A recently published review by Muir et al. (2014) highlights breeding as an effective tool for improving poultry welfare and eliminating the need for beak trimming.

## DISCUSSION

This review summarizes existing knowledge about rearing-related risk factors for poor welfare and productivity in laying hens. On this basis the following recommendations can be made.

- In countries in which beak trimming is still permitted, rearers should apply best practice. This would involve beak trimming using a gentler method (for example IR trimming), removal of as little of the beak as possible, and performance of the operation as early as possible. Because of the adverse effects of beak trimming on bird welfare, alternative strategies for reducing feather pecking (listed below) are preferable. In addition it is possible to reduce the need for beak trimming by using a hybrid that functions better than others in loose housing systems.
- Rearers should house pullets in environments that are as similar as possible to the production environment to ensure optimal utilization of resources and to avoid frustration and associated injurious pecking.
- Starting shortly after hatching, pullets should have constant access to an appropriate substrate throughout both the rearing and production phases. For adult laying hens housed in furnished cages, the industry as a whole should address the common practice of infrequent substrate provision in order for substrate provision to pullets to be effective in reducing problems with feather pecking. The substrate provided should be of a quality similar to that which the birds will have access to as adults.
- Perches should be provided from 7 days of age at the latest to ensure optimal perch use in the adult birds.
- Pullets should be fed mashed or crumbled feed, and the provision of pellets should be avoided.
- Although research on the subject is sparse, limitation of stocking density in loose housing systems at the end of the rearing period to 14 light-strain pullets/m^2^ or 11 heavy-strain pullets/m^2^ may be an advantage in terms of bird welfare.
- Studies of light intensity suggest an optimum of between 5 and 10 lux, because higher lighting intensities increase injurious pecking.
- During rearing, the longest possible photoperiod should be used that still allows control over the start of lay.
- Birds intended for production at organic farms should have had access to outdoor areas during rearing.
- Rearers should avoid large variation in the sound levels as well as high sound levels.
- Rearers should avoid high ammonia and carbon dioxide concentrations.
- Transfer of pullets from the rearing to the laying facility should take place before the pullets reach 16 weeks of age.
- Staff should seek to improve their competence through collaboration with skilled professionals.
- In addition to the above, selection of commercial genetic lines with a low propensity for abnormal behavior and with moderate levels of fearfulness should continue.

This review identifies a number of topics that are not well understood. Despite ongoing debate in many European countries regarding whether beak trimming should be practiced or not, no recent study clearly documents the benefits of, or necessity for, this practice. This review presents convincing evidence of the advantages associated with rearing hens in an environment similar to the one in which they will later live as adults. However, the lack of detailed knowledge regarding the mechanisms underlying the detrimental effects of rearing in one system and producing in another means that further study is required. As an example, the basis of the contradictory welfare consequences (Tahamtani et al., 2014) of combining rearing in an aviary system with production in furnished cages is poorly understood. The welfare effects of age at transfer between rearing and production facilities should also be studied further. Relative to their apparent importance for both welfare and productivity, the welfare consequences of growth rate during rearing, as well as body weight and uniformity at the initiation of lay, have also been sparsely studied. Other neglected areas of research are how welfare during the rearing and laying phases is affected by bird density, feeder distribution, and lighting (light source, flickering, and spectrum) during rearing.
